# Juno Plasma Wave Observations at Ganymede

**DOI:** 10.1029/2022GL098591

**Published:** 2022-12-12

**Authors:** W. S. Kurth, A. H. Sulaiman, G. B. Hospodarsky, J. D. Menietti, B. H. Mauk, G. Clark, F. Allegrini, P. Valek, J. E. P. Connerney, J. H. Waite, S. J. Bolton, M. Imai, O. Santolik, W. Li, S. Duling, J. Saur, C. Louis

**Affiliations:** ^1^ Department of Physics and Astronomy University of Iowa Iowa City IA USA; ^2^ The Johns Hopkins University Applied Physics Laboratory Laurel MD USA; ^3^ Southwest Research Institute San Antonio TX USA; ^4^ Department of Physics and Astronomy University of Texas at San Antonio San Antonio TX USA; ^5^ Goddard Space Flight Center Greenbelt MD USA; ^6^ Department of Electrical Engineering and Information Science National Institute of Technology (KOSEN), Niihama College Niihama Japan; ^7^ Department of Space Physics Institute of Atmospheric Physics of the Czech Academy of Sciences Prague Czechia; ^8^ Faculty of Mathematics and Physics Charles University Prague Czechia; ^9^ Center for Space Physics Boston University Boston MA USA; ^10^ Institute of Geophysics and Meteorology University of Cologne Cologne Germany; ^11^ School of Cosmic Physics, DIAS Dunsink Observatory Dublin Institute for Advanced Studies Dublin Ireland

**Keywords:** Ganymede, plasma waves, Juno

## Abstract

The Juno Waves instrument measured plasma waves associated with Ganymede's magnetosphere during its flyby on 7 June, day 158, 2021. Three distinct regions were identified including a wake, and nightside and dayside regions in the magnetosphere distinguished by their electron densities and associated variability. The magnetosphere includes electron cyclotron harmonic emissions including a band at the upper hybrid frequency, as well as whistler‐mode chorus and hiss. These waves likely interact with energetic electrons in Ganymede's magnetosphere by pitch angle scattering and/or accelerating the electrons. The wake is accentuated by low‐frequency turbulence and electrostatic solitary waves. Radio emissions observed before and after the flyby likely have their source in Ganymede's magnetosphere.

## Key Points

- Whistler‐modes, electron cyclotron harmonics, upper hybrid band and radio emissions are detected in and near Ganymede's magnetosphere
- Three distinct regions in and near the magnetosphere are observed, wake, nightside, and dayside
- The dayside ionosphere exhibits higher and more variable electron densities than does the nightside

## 1 Introduction

Just prior to the end of its prime mission, Juno flew by Ganymede (Hansen et al., 2022). The flyby takes advantage of Juno's advanced instrument complement to study details of the plasma, energetic particles and fields involved in the interaction between Ganymede's and Jupiter's magnetospheres. This paper focuses on plasma waves in Ganymede's magnetosphere.

Galileo plasma wave and magnetic field measurements revealed the existence of Ganymede's magnetosphere during its first flyby of the moon (Gurnett et al., 1996; Kivelson et al., 1996). Additional Galileo studies included six close flybys (Shprits et al., 2018). The plasma wave observations showed a variety of emissions commonly associated with planetary magnetospheres, including whistler‐mode emissions, electron cyclotron harmonics, a band at the upper hybrid frequency, broadband noise bursts at the magnetopause, and even radio emissions emanating from the moon's magnetosphere (Gurnett et al., 1996; Kurth et al., 1997).

**Figure 1:**
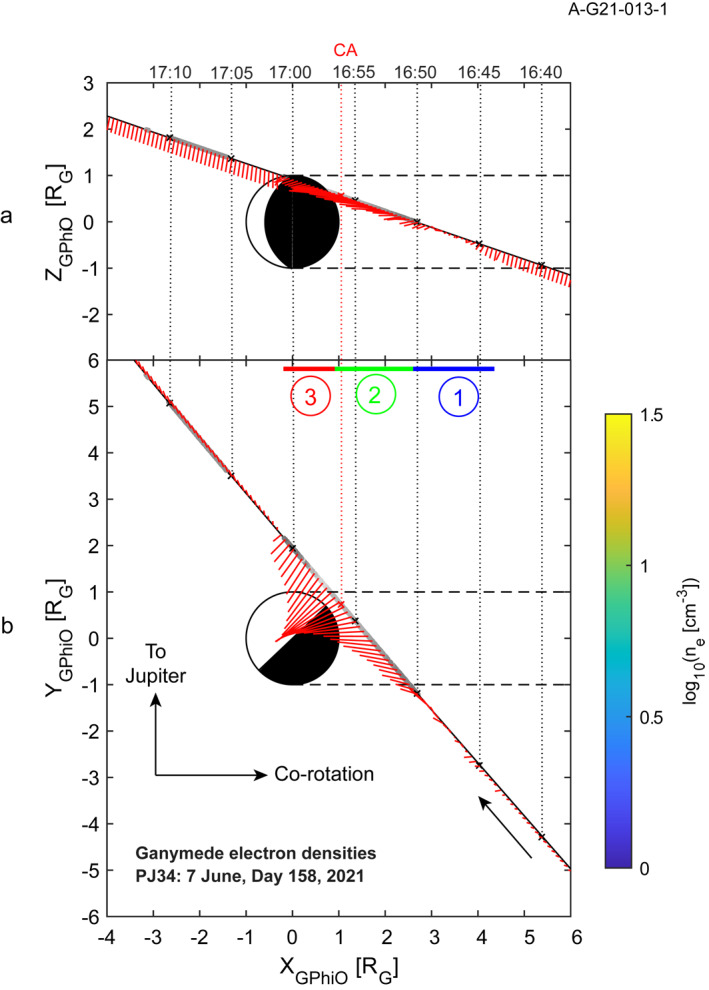
A summary of the geometry of the Juno flyby in co‐rotational coordinates (a) projected into the *z*‐*x* plane and (b) projected into the *y*‐*x* plane. The red ‘whiskers’ indicate the component of the magnetic field as measured by the Juno Magnetometer in that plane. The colors indicated on the trajectory are the log of the electron density inferred from *f*_*uh*_. The blue, green, and red bars are regions identified by differing characteristics in the plasma wave spectrum.

The Juno spacecraft executed a close flyby of Ganymede at 16:56 on 7 June, day 158, 2021 with a closest approach altitude of 1046 km. The trajectory approached Ganymede over its leading hemisphere or downstream from the moon relative to the co‐rotational flow of Jupiter's magnetospheric plasma. The trajectory projected into the z‐x and y‐x planes is shown in Figure 1 using Ganymede‐centered co‐rotational coordinates (sometimes referred to as G_PhiO_). The +*z* axis is parallel to Jupiter's rotational axis and the +*x* axis is parallel to the nominal co‐rotational plasma flow. The +*y* axis is in the direction of Jupiter. The radius of Ganymede (R_G_) is 2,631.2 km. The blue, green, and red bars denoted with the numbers 1, 2, and 3 identify regions observed in Ganymede's magnetosphere and will be used to organize the discussion of the Waves observations.

The Juno Waves instrument is described by Kurth et al. (2017). For the purposes of this paper the instrument covers the frequency range of 40 Hz to 40 MHz using a short electric dipole antenna and from 40 Hz to 20 kHz using a search coil magnetometer. The effective axis of the electric antenna is in the spacecraft *y*‐direction while the search coil's sensitive axis is parallel to the spacecraft *z* axis (spin axis). The response of the electric antenna is given in Sampl et al. (2016, 2021). For the observations included in this paper, three receivers were used. The low‐frequency receiver high band (LFRH) is used for the electric component of the highest frequency waves (10–150 kHz). Two receivers (LFRL and LFRB) covering 40 Hz to 20 kHz are used for simultaneous electric and magnetic spectral measurements. The LFRH (LFRL/B) receiver obtains digitized waveforms at 16‐b resolution sampled at 375 ksps (50 ksps) consisting of 6,144 samples each, once per second. For continuous survey observations, these waveforms are Fourier transformed onboard with the resulting components combined to yield quasi‐logarithmically spaced spectra with ∼18 channels per decade of frequency. The waveforms can also be downlinked forming a burst mode data product.

## 2 Observations

**Figure 2:**
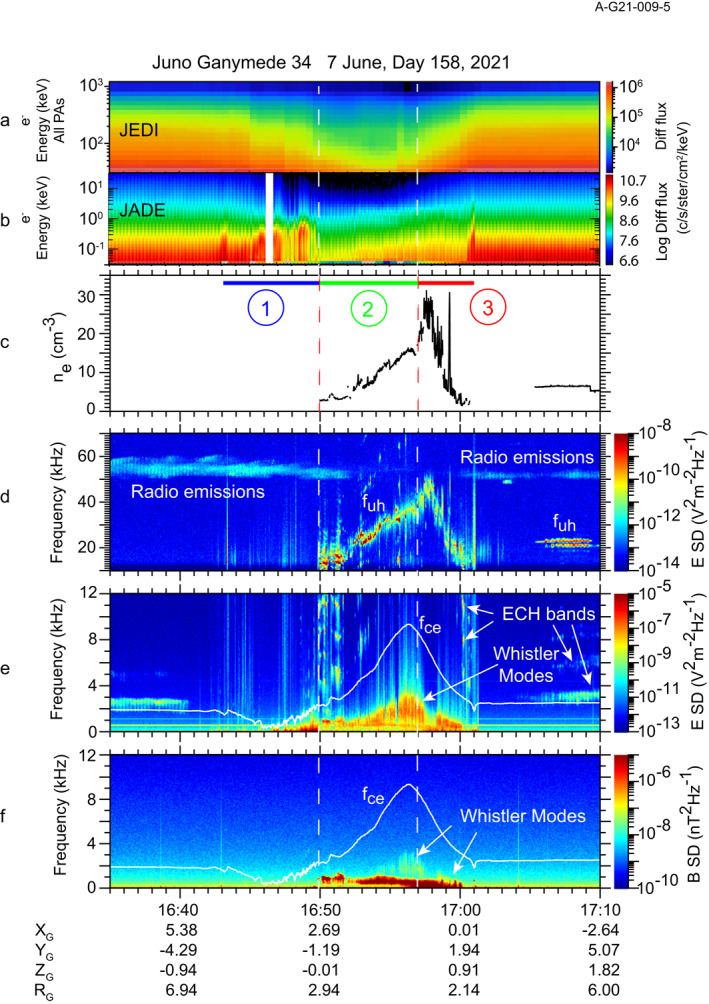
A summary of electron observations and radio and plasma wave observations from the Juno Ganymede flyby. (a) Energetic electron energy‐time spectrogram from the JEDI instrument. (b) Plasma electron energy‐time spectrogram from the JADE instrument. (c) Electron densities inferred from the *f*_*uh*_ band shown in (d). (d) Electric field spectrogram from 10 to 70 kHz. (e) Electric field spectrogram from 40 Hz to 12 kHz. (f) Magnetic field spectrogram from 40 Hz to 12 kHz. The white trace superposed in panels e and f is *f*_*ce*_.

Figure 2 provides an overview of the Waves burst mode observations near Ganymede in context with electron energy spectra. Panel a shows the energy‐time spectrogram for electrons above about 30 keV (Clark et al., 2022) from the Jovian Energetic Particle Detector (JEDI) (Mauk et al., 2013). The lower energy electron spectra (<32 keV) (Allegrini et al., 2022) from the Jovian Auroral Distributions Experiment (JADE) (McComas et al., 2013) are given in panel b. Panel c is a plot of the electron density determined from the frequency of the upper hybrid resonance *f*_*uh*_ band *f*_*uh*_^2^ = *f*_*pe*_^2^ *+ f*_*ce*_^2^ seen in the spectrogram from the LFRH band of the Waves receiver in panel d. Here *f*_*pe*_ is the electron plasma frequency which is related to the electron number density by *f*_*pe*_ [Hz] = 8980√*n*_*e*_ [cm^−3^] and electron cyclotron frequency *f*_*ce*_ [Hz] ≈ 28|**B**| [nT] where |**B**| is obtained by the Juno Magnetometer (Connerney et al., 2017). Bands at *f*_*uh*_ are also seen outside of Ganymede's magnetosphere prior to the flyby (not shown) giving densities of 5–6 cm^−3^ and after 17:05 near 22 kHz giving an electron density of 6.5 cm^−3^ with densities up to about 12 cm^−3^ somewhat later. Panels e and f of Figure 2 show frequency‐time spectrograms of the electric and magnetic fields up to 12 kHz, respectively, from the LFRL and LFRB receivers. Superposed on panels e and f is a white line representing *f*_*ce*_.

Panels d–f in Figure 2 show two very different intervals associated with Ganymede. From ∼16:43 to 16:50 is Region 1 accentuated by broadband bursty electrostatic emissions. Examination of the waveforms shows occasional electrostatic solitary waves (ESWs) that are found with low frequency turbulence observed in this wake region. Some examples of the waveforms are provided in Figure S1 in Supporting Information S1. One can tell from the *f*_*ce*_ overlaid in Figures 2e and 2f that the magnetic field is weak and variable. We identify Region 1 as a wake whereas the interval from 16:50 to 17:00 is Ganymede's magnetosphere based on a change in composition and magnetic field (Allegrini et al., 2022; Romanelli et al., 2022). The outbound magnetopause crossing, near 17:01 is marked with ESWs. We further subdivide the magnetosphere into two regions (identified as 2 and 3) below.

Early and late in Figure 2d is a band of radio emissions between about 50 and 60 kHz. Gurnett et al. (1996) and Kurth et al. (1997) identified similar emissions as originating in Ganymede's magnetosphere. There are several lines of evidence that suggest the emissions in Figure 2, also, are from Ganymede. First, shown in Figure S2 in Supporting Information S1, a long‐term display of emissions in the kilometric wavelength range leading up to the Ganymede flyby shows a number of features that appear with a period near 10 hr, reflecting Jovian radio emissions beamed as Jupiter rotates every ∼9h 55 m. The radio emissions near Ganymede do not fit into these periodic patterns. Second, these emissions seem to be noticeably more intense than the earlier emissions, even considering a 1/r^2^ intensity relationship as Juno approaches Jupiter. Third, the direction‐of‐arrival of these emissions using the rotating dipole technique (Imai et al., 2017; Kurth et al., 1975) is inconsistent with a source near Jupiter. Finally, the frequency of the emissions is similar to the peak frequency of upper hybrid emissions observed by Juno, allowing for the conversion of electrostatic waves to the ordinary mode radio waves by a mode conversion mechanism (cf. Fung & Papadopoulos, 1987; Jones, 1980; Oya, 1971) even though Juno did not appear to cross the source(s) directly. These characteristics are similar to those noted by Gurnett et al. (1996) and Kurth et al. (1997).

**Figure 3:**
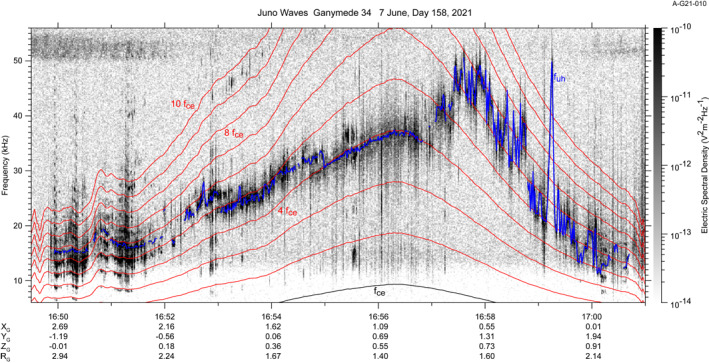
A detail of the electron cyclotron harmonic emissions from ∼10 to 55 kHz. The red traces are harmonics of *f*_*ce*_. The emission identified as *f*_*uh*_ is highlighted in blue.

The other phenomenon in Figure 2d is a set of electron cyclotron harmonic emissions (ECH) in Ganymede's magnetosphere rising from about 10 kHz at 16:50 to a peak near 50 kHz at 16:58 and decreasing in frequency until they disappear near 17:00. These are shown in greater detail in Figure 3. Before 16:52, there are multiple ECH bands extending to the 10th harmonic or even higher. Between 16:52 amd 16:57, there are typically only two intense bands. We have highlighted in blue the upper hybrid resonance band. Hubbard and Birmingham (1978), Kurth et al. (1979) and Kurth (1982) showed that the ECH band containing *f*_*uh*_ is typically the most intense. Hence, particularly for the Juno observations after about 16:52, we can explain the spectrum by the variation of *f*_*uh*_ between the 4th and 6th electron cyclotron harmonics. The apparent abrupt changes in density near 16:53, 16:54, and 16:55 are due to *f*_*uh*_ moving from one ECH band to another and are likely more abrupt than the true change in density. Prior to 16:52, it is difficult to be sure that we have selected the correct band as that including *f*_*uh*_. Our method was to select the most intense band based on earlier work, such as Kurth et al. (2015).

A remarkable feature of the resulting electron density profile in Figure 2c is the dual character of the curve. In Region 2 the density increases to a local maximum near closest approach quite smoothly. However, after 16:57 (Region 3), the density jumps by a factor of ∼two and exhibits large fluctuations with time. Clearly, these two different characters are suggesting two significantly different regions in Ganymede's magnetosphere. Another remarkable feature of the density profile (observed most readily in Figure 3 at 16:59:15) is a very sharp, well‐defined spike, peaking at 30 cm^−3^ and having a width of about 8 s. At Juno's Ganymede‐relative speed of 18.5 km/s, the structure has a width of ∼150 km.

**Figure 4:**
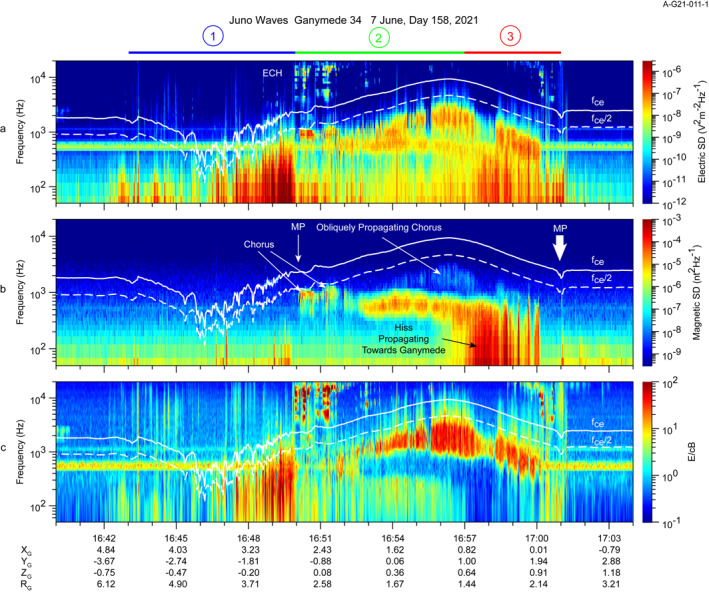
Details of the electric and magnetic spectra observed in Regions 1, 2, and 3 using a logarithmic frequency scale. (a) Electric field spectrogram. (b) Magnetic spectrogram. (c) E/cB versus frequency and time; values near one indicate electromagnetic emissions. Ratios >> 1 indicate electrostatic or quasi‐electrostatic emissions.

Turning, now to the lower frequencies, shown in Figures 2e and 2f, we discuss primarily whistler mode emissions. Between ∼16:50 and 17:00 at frequencies below *f*_*ce*_ are emissions we recognize as whistler modes since they are below both *f*_*ce*_ and *f*_*pe*_. This interval includes Regions 2 and 3 and begins as Juno leaves the wake region at the inbound magnetopause crossing and ends near the outbound magnetopause crossing. Figure 4 shows these emissions using a log frequency scale and adds a computation of the electric to magnetic field strength E/cB. Figure 4, panels a and b, show the electric and magnetic spectra up to 20 kHz with *f*_*ce*_ superposed by the solid white line and *f*_*ce*_/2 using the dashed white line. The magnetopause crossing times are from Romanelli et al. (2022) with the thick arrow at the outbound one indicating the extended magnetopause discussed in that paper. Between 16:50 and 16:52, relatively narrowband and bursty electromagnetic emissions are seen just below *f*_*ce*_/2.

From 16:52 in Region 2 until about 16:57, there are two band‐limited whistler‐mode emissions centered near 2 kHz and several hundred Hz. Expanded frequency‐time spectra are consistent with chorus (Figure S3 in Supporting Information S1), although clearly distinct rising or falling tones are difficult to identify given the instrument duty cycle. Beginning around 16:57 (Region 3) the emission becomes broader in frequency, extending down to the 40‐Hz lower limit of the Waves instrument and suggests a hiss‐like character. At the same time, the upper chorus band is attenuated above several hundred Hz. The time of this spectral change coincides approximately with the abrupt change in the electron density and its transition from relatively smooth to highly variable.

Figure 4c examines the electrostatic versus electromagnetic character of the waves. An electromagnetic wave would be expected to have an E/cB ratio of near 1, whereas electrostatic waves would have large ratios. For example, the low‐harmonic ECH waves between 16:50 and 16:51 show a large E/cB ratio as would be expected for these electrostatic emissions that only appear in panel a. The bursty chorus emissions below *f*_*ce*_ between 16:50 and 16:52 have an E/cB ratio near 1 suggesting electromagnetic waves propagating nearly parallel to **B**. The two emission bands after about 16:52 show starkly different ratios of E/cB. The upper band from 16:52 to near 17:00 is quasi‐electrostatic, showing a large E/cB whereas the band below about 1 kHz is more clearly electromagnetic. That the lower frequency band appears to have a E/cB ratio of less than one reflects the fact that the electric antenna rotates and is not always aligned with the wave electric field, hence, the electric field is underestimated. The quasi‐electrostatic nature of the band above 1 kHz is likely due to whistler mode emissions propagating obliquely near the resonance cone whereas the lower frequency electromagnetic band likely has a wave‐normal angle nearly parallel to **B**. The broadband hiss feature appearing near 16:57 and later (Region 3) is clearly electromagnetic in nature.

Near 16:57:30 the obliquely propagating band shows a distinct decrease in amplitude, shortly after the change in character of the electron density profile. This band recovers in intensity, somewhat, about a minute later. The decrease in amplitude of the obliquely propagating hiss accompanies the appearance of the more nearly parallel‐propagating hiss.

When there is a substantial component of Jupiter's magnetic field parallel to the spacecraft x axis, the phase relation between the electric and magnetic signals from the dipole antenna and search coil can be used to determine a component of the wave's Poynting flux, **S**, either parallel or anti‐parallel to the magnetic field (Kolmasova et al., 2018; Mosier & Gurnett, 1969;). In this case a parallel (anti‐parallel) component of **S** would indicate waves propagating toward (away from) Ganymede. Later in the interval, the orientation of **B** is favorable for such determinations. The whistler mode hiss observed primarily in Region 3 exhibits a component of the Poynting vector parallel to **B**, hence, toward Ganymede. This would imply that the electrons generating these waves are moving away from the moon, assuming first order cyclotron instability. An example of the phase analysis supporting this result is given in Figure S4 in Supporting Information S1.

## 3 Discussion

The plasma wave observations during the Juno flyby on orbit 34 reveal three distinct regions associated with Ganymede's magnetosphere. In time order, the first is best described as a wake region, extending from near 16:43 to the magnetopause at 16:50. The region is characterized by bursty emissions, mostly electrostatic but with some low‐frequency magnetic components seen most readily in Figure 4b. This turbulence may be the upper extension of Alfvén waves related to the lower frequency magnetic fluctuations. The magnetic field goes through a minimum in this region. There are no features that can be associated with the plasma frequency or upper hybrid frequency in this region, hence, we have no information on electron density, although the JADE instrument observes compressed, heated plasma in this region and measures electron/heavy ion densities of ∼3 to ∼20 (average ∼7) cm^−3^/∼6 to ∼24 (average ∼15) cm^−3^ here (Allegrini et al., 2022). The bursty emissions are reminiscent of broadband electrostatic noise (Gurnett & Frank, 1977) later determined to be ESWs by Matsumoto et al. (1994). As shown in SI (Figure S1 in Supporting Information S1), there is evidence of ESWs , but likely other modes, as well. Duling et al. (2014) and Jia (2015) suggest Ganymede's wake may be the site of reconnection of fields that originally reconnected on the upstream side of the magnetosphere. Romanelli et al. (2022) and Ebert et al. (2022) argue that evidence for reconnection in the extended upstream magnetopause is observed by Juno and the ESWs observed by Waves (see examples in Figure S5 in Supporting Information S1) supports this. ESWs are an end‐stage of a non‐linear evolution of current instabilities (Matsumoto et al., 1994) and would be expected in the magnetotail and magnetopause regions as they are current‐carrying boundary layers. Jia (2015) suggests this reconnection is intermittent and bursty on the upstream magnetopause and that the reconnection in the tail serves to return magnetic flux and may drive particle acceleration. The resulting dynamics may include depressions in the magnetic field as observed by Juno and heated plasma as reported by the JADE instrument (Allegrini et al., 2022).

The two regions in the magnetosphere are characterized by whistler‐mode and ECH emissions. We distinguished these two regions based on the nature of the observed electron densities (Figure 2c). From 16:50 to 16:57 the densities rise to a peak at ∼15 cm^−3^ at closest approach with only minor variations. In the second region, the densities peak at a factor of two higher, near 30 cm^−3^ and with sporadic variations. Why do these two regions have such different electron density profiles? It seems clear that Juno crossed a boundary near 16:57 between two very different magnetospheric regions that was apparently not observed by Galileo (cf. Eviatar et al., 2001). One possibility is that Juno crossed a region of closed magnetic field lines in Region 2 meaning both ends of the field are in Ganymede. Region 3, then, would be on open field lines, with one foot in Ganymede and the other in Jupiter. Romanelli et al. (2022) suggest a region of closed field lines extending from about 16:54 to 16:58, but neither of these boundaries correspond to the time of the change between the two regions of density character. But, Allegrini et al. (2022), Clark et al. (2022), and Duling et al. (2022) argue no closed field lines were crossed by Juno.

Another possible explanation of the different nature of regions 2 and 3 lies in the fact that Juno crossed from the nightside to the dayside at about this same time. Juno's local time with respect to Ganymede crossed 6 hr at about 16:56:40. Further, modeling by Duling et al. (2022) indicates Juno crossed from magnetic field lines with one foot on Ganymede's nightside to having that foot on the dayside at about the same time. While a higher ionospheric density on the dayside is to be expected, one might question whether the change could be this abrupt. But, since a day on Ganymede is about 7 Earth days, the rotation is not rapid and even near the dawn terminator the dayside ionosphere has had the advantage of solar illumination and photoionization for a comparatively long time. The solar heating may also lead to larger electron temperatures and, therefore, large and maybe irregular transport along field lines, leading to the variable density, but we admit that we have not developed a theory for this.

Buried within the dayside region is a singular spike with a density of 30 cm^−3^ isolated within an 8‐s interval. We've found no complementary observations from either the magnetometer or particle instruments that coincide with this feature. The simplest explanation is that this is a duct. We have also considered that this might be a plume, but so far from the moon, it seems unrealistic to see a plume confined to just ∼150 km. Also, there is apparently no magnetic field fluctuation one might expect with a plume.

The whistler‐mode emissions seen throughout the magnetosphere, are of course, a common feature of planetary magnetospheres (cf. Kurth & Gurnett, 1991). They can be important both for the acceleration of electrons (Thorne et al., 2013) and for pitch‐angle scattering (Li, Ma, Shen, Zhang, et al., 2021). The upper band which appears in Figure 4 as being quasi‐electrostatic is propagating obliquely with a wave‐normal angle near the resonance cone. Li et al. (2016) show that at Earth a simple temperature anisotropy is insufficient to generate obliquely propagating chorus, but that the emission can be caused by a low‐energy beam‐like distribution. But, Santolik et al. (2010) show that highly anisotropic injected electrons can generate oblique, upper‐band chorus. These oblique waves may result in filling the downward (toward Ganymede) loss cone. The obliquely propagating chorus may play a major role in precipitating electrons below a few hundred eV (Li, Ma, Shen, Zhang, et al., 2021), contributing to Ganymede's aurora (Allegrini et al., 2022; Saur et al., 2022). The lower band is electromagnetic in nature, hence, is propagating quasi‐parallel to the magnetic field. This band can be driven by a temperature anisotropy in the electron distribution. The hiss extending to lower frequencies would imply interactions with higher energy electrons (Li, Ma, Shen, & Zhang, 2021).

The plasma and radio waves observed near Ganymede by Juno are reminiscent of those observed by Galileo (Gurnett et al., 1996). One major difference is the extended wake region traversed by Juno that Galileo did not cross. Another is the upper band of obliquely‐propagating chorus observed by Juno which was apparently not observed by Galileo (Shprits et al., 2018). The lower chorus band reported herein with E/cB ∼ 1 is similar to the Galileo chorus observations. The magnetic wave power for Juno at 700 Hz is ∼10^−3^ nT^2^, similar to the median value for chorus in Ganymede's magnetosphere reported by Shprits et al. (2018). The difference in flyby trajectories may be the reason for the additional, quasi‐electrostatic band in the Juno observations. The Juno observations are also supported by particle measurements with good pitch angle coverage and composition measurements. The Juno wave measurements, with simultaneously recorded electric and magnetic waveforms allow, in some cases, the determination of the sign of a component of the Poynting flux, that is, parallel or anti‐parallel to Ganymede's magnetic field.

## 4 Conclusions

Juno executed a flyby (1,046 km) of Ganymede before its 34th perijove. The Juno Waves instrument measured plasma waves associated with Ganymede's magnetosphere, including whistler‐mode chorus and hiss, ECH bands including a band at *f*_*uh*_, and ESWs associated with Ganymede's magnetopause and wake. The *f*_*uh*_ provides a measure of *n*_*e*_, revealing two distinct regions in the magnetosphere: one with smoothly increasing density to a peak near 15 cm^−3^ and a more disturbed region with peak densities near 30 cm^−3^ during the outbound passage. The boundary between these two regions occurs as Juno moves from the nightside to dayside ionosphere, perhaps providing the additional electron density and even turbulence observed. The Juno trajectory approached Ganymede on its corotational wake hemisphere (orbital leading hemisphere). Here, the Waves instrument found broadband bursts of plasma waves including ESWs demarcating entry into an extended wake with continuing bursty emissions up to the magnetopause. Multiple ECH emissions are observed just inside the magnetopause evolving into just one or two bands near *f*_*uh*_. These bands were observed to just beyond a local peak in the density at closest approach before a much more disordered set of emissions at the *f*_*uh*_. Below *f*_*ce*_ whistler‐mode emissions are observed. These include both quasi‐parallel and obliquely propagating chorus as well as quasi‐parallel hiss. ESWs were also observed at the outbound magnetopause crossing. A narrowband radio emission observed near 50 kHz, before and after the flyby, is likely generated in Ganymede's magnetosphere via mode conversion from electrostatic waves at frequencies related to *f*_*pe*_.

## Supporting information

Supporting Information S1

## Data Availability

Waves burst data used herein is found in the Planetary Data System (PDS) https://pds.nasa.gov at https://doi.org/10.17189/1522461. Waves survey data are at https://doi.org/10.17189/1520498. Juno Magnetometer data are at https://doi.org/10.17189/1519711. JEDI data are at https://doi.org/10.17189/1519713. JADE data may be found at https://doi.org/10.17189/1519715.
