# Supplementary material for: Juno Plasma Wave Observations at Ganymede
Source: Geophys Res Lett. 2022 Dec 12;49(23):e2022GL098591. doi: 10.1029/2022GL098591 (PMC10078157; doi:10.1029/2022GL098591)
Supplement: Supplementary file 1 — Supporting Information S1 [file GRL-49-0-s001.pdf]

AGU PUBLICATIONS

Geophysical Research Letters

Supporting Information for

Juno Plasma Wave Observations at Ganymede

W. S. Kurth[1], A. H. Sulaiman[1], G. B. Hospodarsky[1], J.D. Menietti, B. H. Mauk[2], G. Clark[2], F. Allegrini[3,4], P. Valek[3], J. E. P. Connerney[5], J. H. Waite[3], S. J. Bolton[3], M. Imai[6], O. Santolik[7,8], W. Li[9], S. Duling[10], J. Saur[10], C. Louis[11]

[1]Department of Physics and Astronomy, University of Iowa, Iowa City, IA USA. [2]The Johns Hopkins University Applied Physics Laboratory, Laurel, MD, USA. [3]Southwest Research Institute, San Antonio, TX, USA. [4]Department of Physics and Astronomy, University of Texas at San Antonio, San Antonio, Texas, USA [5]Goddard Space Flight Center, Greenbelt, MD, USA. [6]Department of Electrical Engineering and Information Science, National Institute of Technology (KOSEN), Niihama College, Niihama, Ehime, Japan. [7]Department of Space Physics, Institute of Atmospheric Physics of the Czech Academy of Sciences, Prague, Czechia. [8]Faculty of Mathematics and Physics, Charles University, Prague, Czechia. [9]Center for Space Physics, Boston University, Boston, MA, USA. [10]Institute of Geophysics and Meteorology, University of Cologne, Cologne, Germany. [11]School of Cosmic Physics, DIAS Dunsink Observatory, Dublin Institute for Advanced Studies, Dublin, Ireland.

Contents of this file

Figures S1 to S5

Introduction

The supporting information supplied here provides observations used to support some of the statements in the main manuscript. Below we describe the intent of each of the supporting illustrations.

Text S1.

Figure S1 provides evidence for low-frequency waves along with electrostatic solitary waves in Region 1 (wake).

Text S2.

Figure S2 provides the Ganymede observations in the range of 30 to 130 kHz in context with Jovian radio emissions in order to suggest that the radio emissions observed between 50 and 60 kHz shortly before and after the Ganymede flyby are likely generated in or at Ganymede's magnetosphere, as opposed to a source associated with Jupiter's plasma torus.

Text S3.

While the duty cycle of the Waves burst mode below 20 kHz (~116 ms/s) makes it difficult to observe discrete rising or falling tones with times scales of order 1 second, indicative of chorus, the bursty narrowband nature of these emissions suggests they are lower-band chorus. Figure S3 shows high resolution spectrograms that demonstrate the bursty nature of the band-limited whistler-mode emissions in Ganymede's Regions 2 and 3. There is also a suggestion of rising and falling tones in these displays. Hence, we suggest these are bands of chorus as opposed to broadband hiss with little temporal variation.

Text S4.

Figure S4 provides the elements of an analysis of the phase relation between the electric and magnetic components in selected Ganymede whistler-mode emissions. In this analysis the phase relationship can be used to determine one component of the Poynting flux direction, either parallel or anti-parallel to the static magnetic field as described in Kolmasova et al. (2018). These show that the low-frequency hiss appears to propagate rather uniformly with a component in the direction of Ganymede (parallel to B). Since the magnetic component of the upper chorus band is weak, the results are somewhat unreliable for this band but appear to show elements propagating in opposite directions at times.

Text S5.

Figure S5 provides evidence for electrostatic solitary waves near the outbound magnetopause crossing. This phenomenon is associated with an electron beam instability (Matsumoto et al. (1994) and in this context would be expected to be the result of beams flowing in the magnetopause.

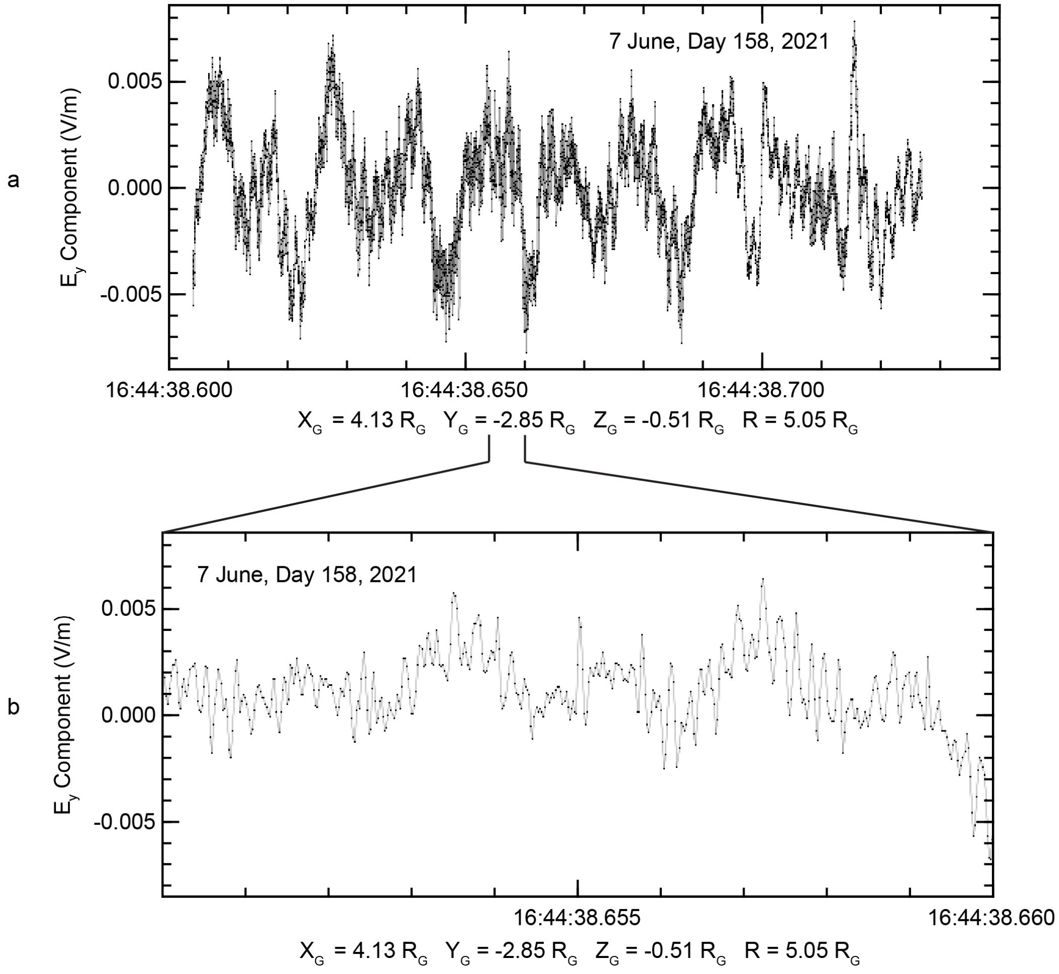

Figure S1. Example waveforms from Region 1 in the wake showing the presence of both low-frequency turbulence and electrostatic solitary waves (ESW) shown most clearly in panel b. ESW's are often found in regions carrying currents and/or beams of electrons.

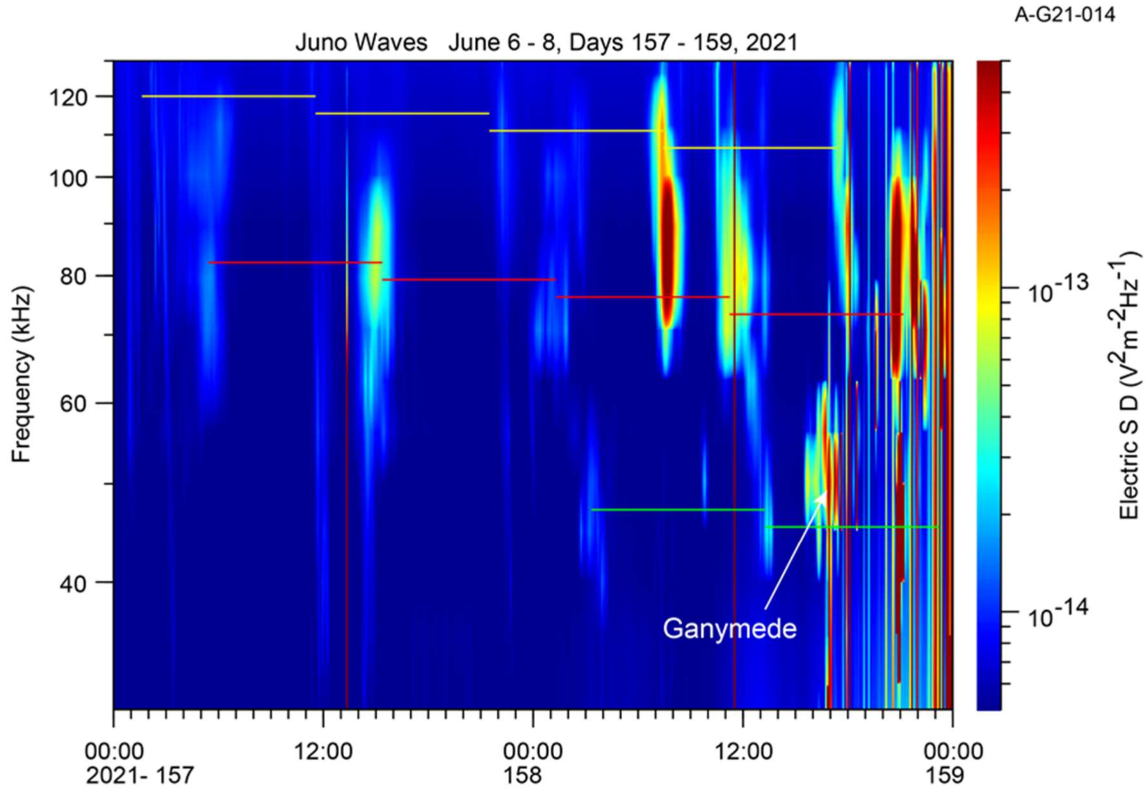

Figure S2. A frequency-time spectrogram showing electric field data from 30 to 130 kHz for a two-day interval, including the Ganymede flyby. The yellow, red, and green horizontal bars are 9 h, 55 m long and are positioned to allow one to see the rotational periodicity of many of the emissions observed in the several tens kHz frequency range. While one can see a suggestion of the expected periodicities in most of these emissions, those near Ganymede do not appear to have such a relationship with earlier events. Also, the Ganymede emissions appear brighter and not suggestive of an r^{-2} relation to a source located near Jupiter. The broadband bursty emission in the last ~6 hours of this interval are thought to be related to dust impacts often seen on approach to Jupiter.

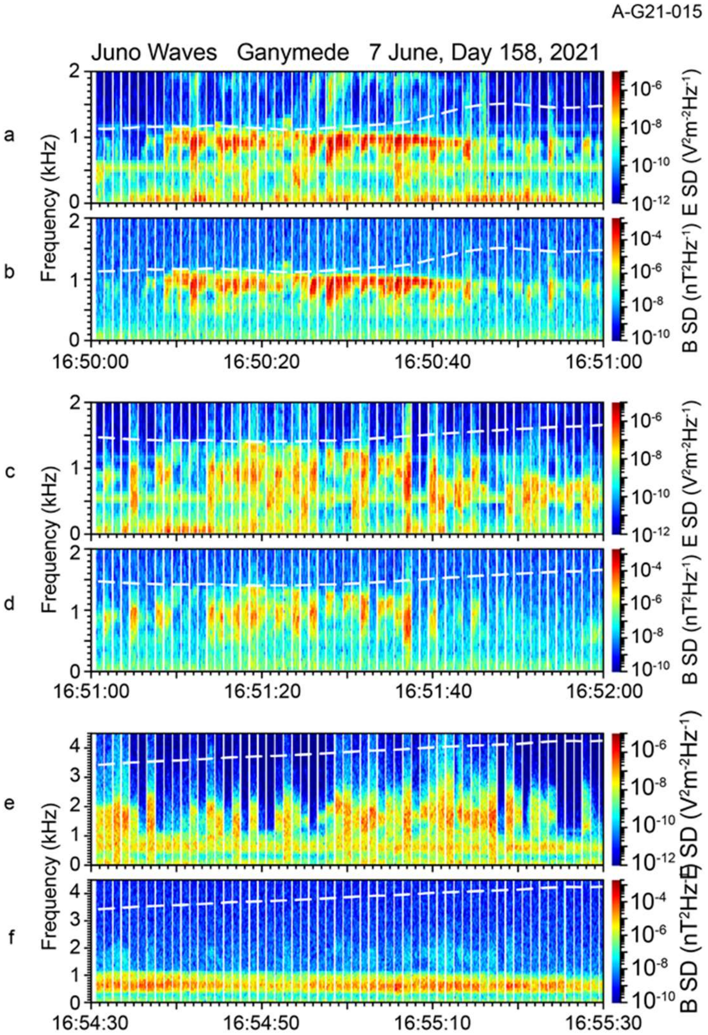

Figure S3. Examples of whistler mode emissions in Ganymede's magnetosphere providing evidence of discrete rising and/or falling tones. Each set of panels shows electric and magnetic spectral densities vs. frequency and time for three minute-long intervals in Region 2. The dashed white line is $f_{ce}/2$. The Waves burst mode data are acquired in this frequency range for ~123 ms every second. In this display we have expanded each 123-ms burst capture to nearly fill each second of the time axis (that is, the time axis of the spectrograms is non-linear). There are narrow white vertical lines indicating the break between one capture and the next. In each case, there are brief, discontinuous emissions with some indication of frequency drift within the 123-ms intervals, although this is not always the case. We suggest these emissions are more consistent with discrete chorus emissions than whistler-mode hiss.

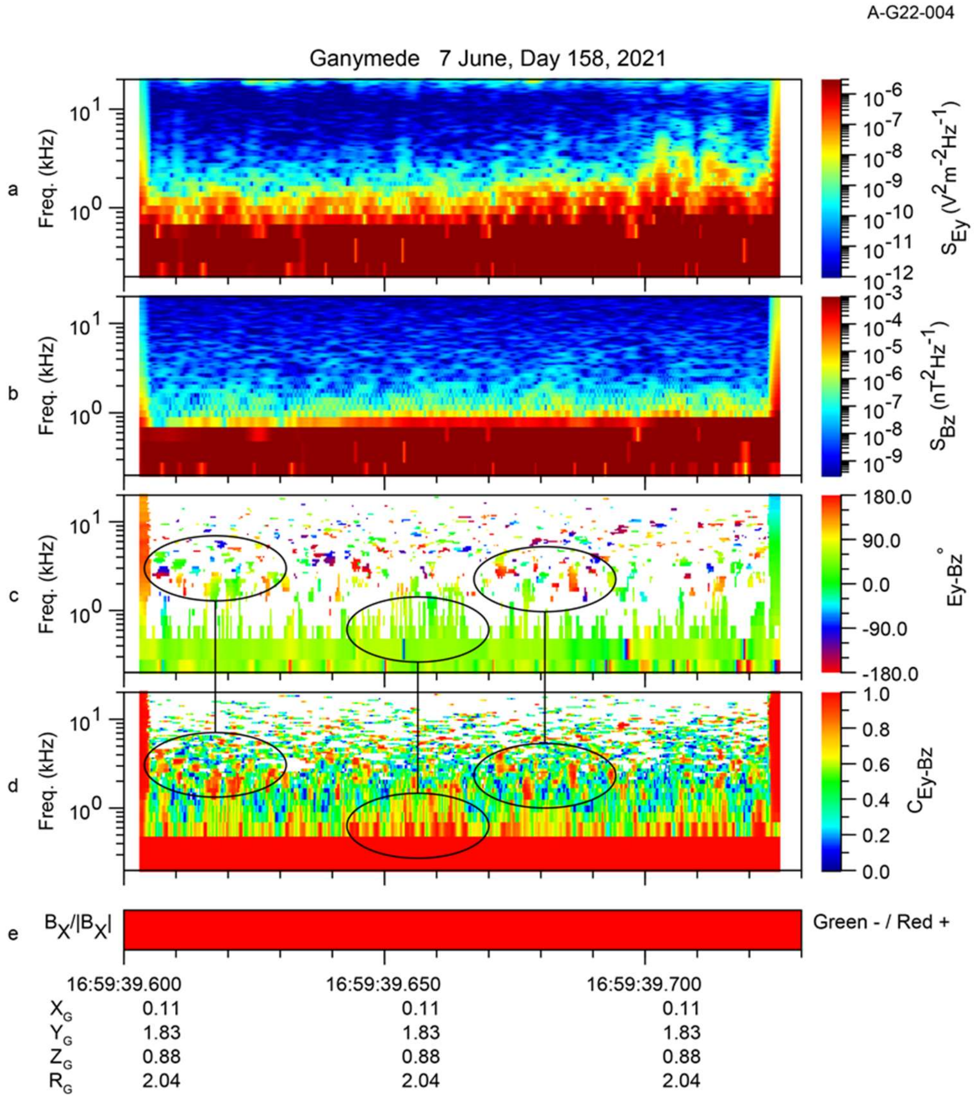

Figure S4. Analysis of the phase correlation (panel c) between the y component of the electric field (panel a) and z component of the magnetic field (panel b), each in spacecraft coordinates. Panel d provides the coherence in the E and B signals; only those frequencies and times with high coherence (red) should be considered when interpreting the phase in panel c. Panel e gives the sign of the static magnetic field in the spacecraft x direction, important in understanding the phase. In this case, small phase differences, for example below 1 kHz, when the Bx component is positive indicates a component of the Poynting flux parallel to B, that is, in the direction of Ganymede. This interval shows hiss propagating with a component of the Poynting vector parallel to B and towards Ganymede. The band near 2 kHz is the quasi-electrostatic chorus band discussed in Figure 4 in the main text. Since the magnetic component is weak, the phase analysis is somewhat unreliable, and shows mixed evidence of propagation towards/away from Ganymede.

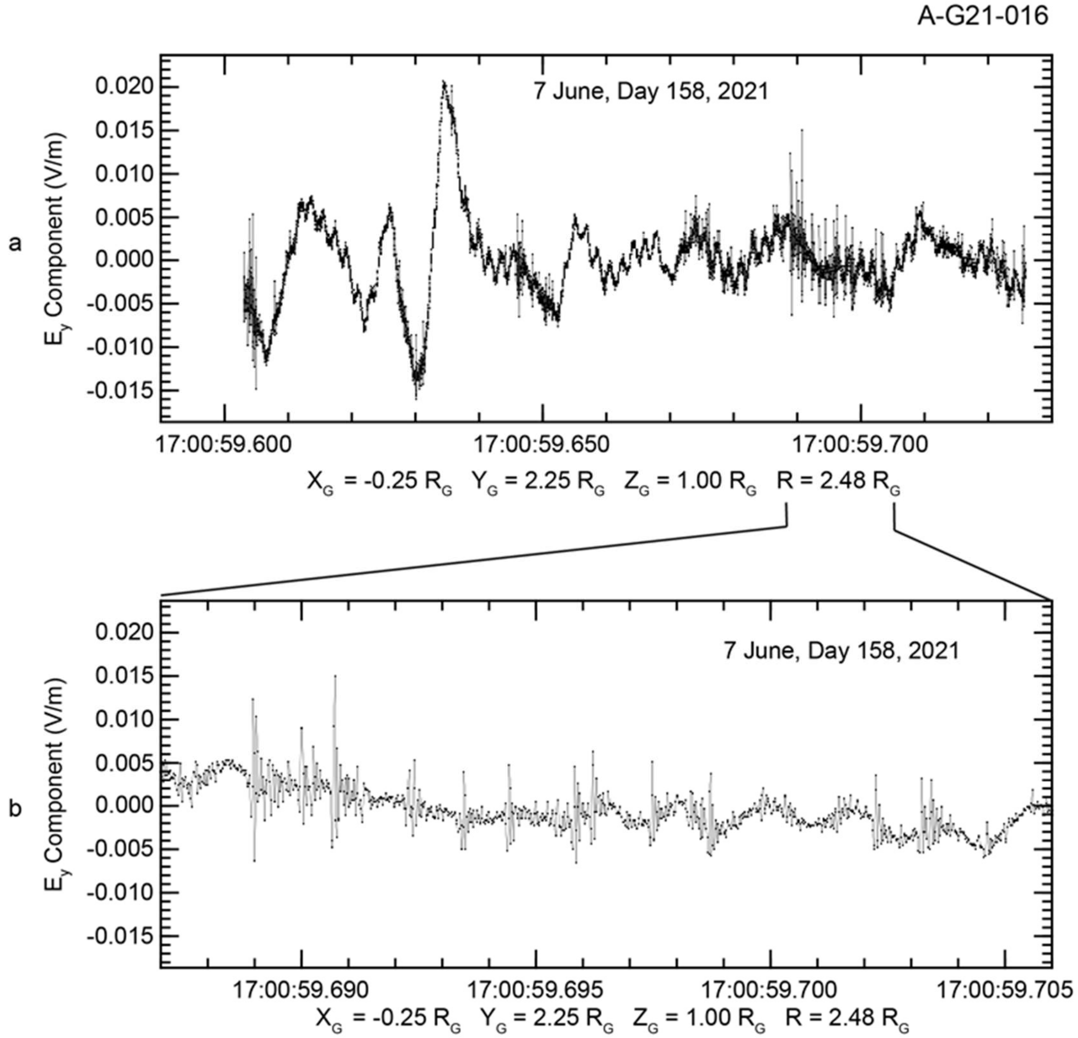

Figure S5. Electric field waveforms obtained in the vicinity of the outbound magnetopause crossing. (a) An electric waveform lasting ~120 ms beginning shortly after 17:00:59.6 UT within the extended upstream magnetopause identified by Romanelli et al. (this issue). (b) An expanded segment of the waveform in panel a. The impulses seen particularly well in panel b are numerous electrostatic solitary waves (ESWs). The Fourier transform of these wave structures accounts for the broadband bursts highlighting the magnetopause in the Waves data. The ESWs can be generated by electron beams, as might be expected in the magnetopause.
